# Determinants of in-hospital death in patients with a thrombus straddling a patent foramen ovale: protocol of a systematic review

**DOI:** 10.12688/f1000research.27622.2

**Published:** 2021-05-05

**Authors:** Amado Jiménez-Ruiz, Palak Shah, Andrew Gibson, Juan Camilo Vargas-González, Luciano A. Sposato

**Affiliations:** 1 Heart & Brain Laboratory, Western University, London, Ontario, N6C1C4, Canada; 2 Department of Clinical Neurological Sciences, Schulich School of Medicine and Dentistry, Western University, London, Ontario, N6C1C4, Canada; 3 Department of Epidemiology and Biostatistics, Schulich School of Medicine and Dentistry, London, Ontario, N6C1C4, Canada; 4 Department of Anatomy and Cell Biology, Schulich School of Medicine and Dentistry, London, Ontario, N6C1C4, Canada; 5 Lawson Health Research Institute, London Health Sciences Centre, London, Ontario, N6C1C4, Canada; 6 Robarts Research Institute, Western University, London, Ontario, N6C1C4, Canada

**Keywords:** Stroke, myocardial infarction, patent foramen ovale, paradoxical embolism, death, mortality, thrombus in transit

## Abstract

**Background: **Thrombi identified on echocardiography at the time of straddling a patent foramen ovale (PFO) constitute a medical emergency with an associated imminent risk of death.  Ischemic stroke (IS) and myocardial infarction (MI) occurring in patients with a thrombus straddling a PFO (TSPFO) may be associated with increased risk of in-hospital death. Variables associated with increased risk of death in women and men may be different. We will perform a systematic review of case reports and cases series of patients with a TSPFO to assess if IS and MI are associated with increased risk of in-hospital death and we will further stratify analyses by sex.

**Methods: **This systematic review will include all case reports and case series of adult patients (18-year-old or older) with echocardiographic or pathological (e.g. at autopsy for older reports) evidence of a TSPFO published between inception and June 30, 2020, in any language. We will search in PubMed and Embase databases. Two reviewers will independently screen titles and abstracts, retrieve full texts, and extract the data in a predesigned form. We will apply a multivariable logistic regression analysis to estimate the association of IS and MI with in-hospital mortality. We will stratify analyses by sex.

**Discussion:** IS and MI in patients with TSPFO could potentially be associated with worse outcomes if they are not timely identified or left untreated.  Both acute IS and MI require specific treatment (e.g. thrombolysis, primary coronary intervention, or mechanical thrombectomy) that may be influenced by the therapy instituted for the TSPFO. Knowing the incidence of acute IS and MI among patients diagnosed with TSPFO and whether they are associated with an increased risk of death would help to improve the management of this medical emergency.

**Protocol registration**: CRD42020216118, PROSPERO.

## Introduction

A patent foramen ovale (PFO) is a common finding, present in 25% of individuals on autopsy ^1^ and frequently reported as an incidental finding on echocardiography studies ^2^. Most patients are asymptomatic, and the presence of a PFO is usually considered clinically irrelevant. However, a thrombus can sometimes be found straddling a PFO in the context of acute pulmonary embolism, deep venous thrombosis, acute respiratory insufficiency, acute coronary syndromes, or acute ischemic stroke (IS) ^3^.

A thrombus straddling a PFO (TSPFO) carries a high risk of impending paradoxical embolism and death ^4^. Most of the evidence on the diagnosis and treatment of TSPFO comes from anecdotal single case reports or small case series ^4^. As such, factors associated with increased risk of death are unknown. Similarly, there is clear guidance on what the best treatment approach is for TSPFO. Paradoxical embolism is a relatively common cause of IS in patients with PFO and could possibly explain the high mortality in patients with a TSPFO ^5^. Although less frequently reported, paradoxical embolism has also been identified as a cause of acute myocardial infarction (MI). Both IS and MI are well recognized causes of death among the general population. We hypothesized that IS and MI identified at the time of the diagnosis of a TSPFO are associated with increased risk of in-hospital death. We will therefore systematically review all case reports and case series of patients with TSPFO published in the medical literature to assess whether IS, MI, and different therapeutics (e.g. anticoagulation, thrombolysis or surgical removal of the TSPFO) are associated with the adjusted risk of in-hospital death. We will also stratify these analyses by sex.

## Methods/design

### Objectives

The primary objective is to assess whether IS and MI at presentation are independently associated with the adjusted risk of in-hospital death in patients with a TSPFO. The secondary objective is to assess whether the association between IS and MI at presentation and the risk of in-hospital death in patients with a TSPFO varies in women and men.

### Study design

This study protocol has been prepared according to the 2015 Preferred Reporting Items for Systematic Reviews, and Meta-Analyses Protocols (PRISMA-P) guidelines. We will use the PRISMA flowchart. We have submitted this systematic review to the International Prospective Register for Systematic Reviews and Meta-analysis (PROSPERO, registration number: CRD42020216118).

### Search strategy

We will search PubMed and Embase databases to identify potentially eligible studies by applying predefined search terms ( Table 1 and Table 2), published from inception to June 30, 2020 in any language.

**Table 1.** PubMed search terms.

Transit[Title/Abstract] AND embol*[Title/Abstract] AND patent[Title/Abstract] Migrat*[Title/Abstract] AND embol*[Title/Abstract] AND patent[Title/Abstract] Transit[Title/Abstract] AND Thromb*[Title/Abstract] AND patent[Title/Abstract] Migrat*[Title/Abstract] AND Thromb*[Title/Abstract] AND patent[Title/Abstract] Travel*[Title/Abstract] AND embol*[Title/Abstract] AND patent[Title/Abstract] Travel*[Title/Abstract] AND Thromb*[Title/Abstract] AND patent[Title/Abstract] Pending*[Title/Abstract] AND embol*[Title/Abstract] AND patent[Title/Abstract] Pending*[Title/Abstract] AND Thromb*[Title/Abstract] AND patent[Title/Abstract] Straddling*[Title/Abstract] AND embol*[Title/Abstract] AND patent[Title/Abstract] Straddling*[Title/Abstract] AND Thromb*[Title/Abstract] AND patent[Title/Abstract] Impending*[Title/Abstract] AND embol*[Title/Abstract] AND patent[Title/Abstract] Impending*[Title/Abstract] AND Thromb*[Title/Abstract] AND patent[Title/Abstract] Impending*[Title/Abstract] AND paradoxical[Title/Abstract] AND patent [Title/Abstract] Floating*[Title/Abstract] AND embol*[Title/Abstract] AND patent[Title/Abstract] Floating*[Title/Abstract] AND Thromb*[Title/Abstract] AND patent[Title/Abstract] Entrapped[Title/Abstract] AND embol*[Title/Abstract] AND patent[Title/Abstract] Entrapped[Title/Abstract] AND Thromb*[Title/Abstract] AND patent[Title/Abstract] Saddl*[Title/Abstract] AND patent[Title/Abstract] Biatrial[Title/Abstract] AND patent[Title/Abstract] Impending paradoxical embol*[Title/Abstract] Transit[Title/Abstract] AND thromb*[Title/Abstract] AND atri*[Title/Abstract] Crossing[Title/Abstract] AND thromb*[Title/Abstract] AND atri*[Title/Abstract] Crossing[Title/Abstract] AND embol*[Title/Abstract] AND atri*[Title/Abstract] Thromb*[Title] AND patent foramen ovale[Title] Pulmonary[Title/Abstract] AND paradoxical[Title/Abstract] Riding[Title/Abstract] AND thromb*[Title/Abstract] AND atri*[Title/Abstract] Riding[Title/Abstract] AND embol*[Title/Abstract] AND atri*[Title/Abstract] Stuck[Title/Abstract] AND thromb*[Title/Abstract] AND atri*[Title/Abstract] Stuck[Title/Abstract] AND embol*[Title/Abstract] AND atri*[Title/Abstract]

**Table 2.** Embase search terms.

Impending paradoxical embolism
Thrombus in transit

### Study selection

Three reviewers will independently screen titles and abstracts and will solve disagreements by consensus (AJR, PS, AG). The same reviewers will thoroughly assess all potentially relevant full texts and will document the reasons for excluding specific publications. We will include studies fulfilling all inclusion criteria and no exclusion criteria. Inclusion criteria will be: (a) case reports or case series; (b) adult patients (≥18 years-old); (c) complete data on in-hospital outcomes (e.g. dead or alive); and (d) complete demographic data (e.g. age and sex) and information on comorbidities, risk factors and acute treatment. Exclusion criteria include: (a) editorial or review articles; (b) duplicate reports; and (c) publications in which data at the patient-level is unavailable. 

### Data extraction

Three independent reviewers will be used for selecting studies through each phase of the review (screening, eligibility, and inclusion meta-analysis). We will create and use a standardized Microsoft Forms data extraction form and Excel spreadsheet, extracting the following data from published reports: study identification (year of publication, first author); study characteristics (number of cases, continent where the study was conducted); patients' characteristics (age, sex), risk factors and comorbidities (obesity, hypertension, diabetes mellitus, hyperlipidemia, known atrial fibrillation, coronary artery disease, obstructive sleep apnea, cancer, chronic kidney disease, deep vein thrombosis, pulmonary embolism, transient ischemic attack, stroke, autoimmune disease); acute diagnoses upon admission (acute MI, acute IS, transient ischemic attack, hemorrhagic stroke, pulmonary embolism, deep vein thrombosis, syncope, peripheral paradoxical embolism, newly diagnosed atrial fibrillation); presenting symptoms (dyspnea, chest pain, palpitations, dizziness, syncope, focal neurological deficit, shock, loss of consciousness or coma, seizures, peripheral embolism); laboratory parameters (D-Dimer value, fibrinogen, brain natriuretic peptide, cardiac troponin); main affected coronary artery in patients with MI; vascular territory involved in patients with cerebrovascular events; organ involved in patients with peripheral embolism; most likely cause of venous thromboembolism (unprovoked, thrombophilia, cancer, trauma, post-operative, immobilization, pregnancy, recent flight, infection, other); cardiac and pulmonary investigations (transthoracic echocardiogram, transesophageal echocardiogram, computed angiography of the lungs, cardiac magnetic resonance imaging, cardiac computed tomography, electrocardiogram); main electrocardiographic findings (S1Q3T3 pattern, sinus tachycardia, other); echocardiographic findings [left ventricular dysfunction, right ventricular dysfunction, right ventricular function not reported, increased pulmonary artery pressure (>20 mmHg), dilated right ventricle, dilated right atrium]; acute treatment (intravenous thrombolysis, surgery, anticoagulation); in-hospital outcomes (full recovery, partial recovery, death); secondary prevention treatment (Aspirin or other antiplatelet agent, vitamin K antagonists, non-specified oral anticoagulants, direct oral anticoagulants, low molecular weight heparin, unfractionated heparin, PFO closure, Inferior vena cava filter); and secondary prevention outcome (no events, recurrent venous thromboembolism, incident/recurrent stroke or transient ischemic attack, recurrent/incident peripheral embolism, recurrent/incident MI, death).

### Data analysis

We will conduct univariate analyses comparing the patients who died or survived during hospital stay. Variables with a p-value of <0.05 and those known to influence death in patients with venous thromboembolism, regardless of their level of significance on univariate in the analysis, will be included in a multivariable logistic regression for in-hospital death. We will use a random intercept model to account for the potential clustering effect of the different decades on outcomes. We will initially include all the potential covariates and we will subsequently perform AIC (Akaike Information Criterion) to select the best model and improve overall accuracy. We will keep sex and age in the model because of being recognized confounders for death. We will conduct all analyses with R version 3.6.2.

### Risk of bias and quality of reports

We will apply the tool originally proposed by Murad *et al.* for assessing the methodological quality and synthesis of case series and case reports ^6^ (domains, selection, ascertainment, causality, and reporting).

### Potential amendments

We do not anticipate any amendment to this review protocol. If an amendment is needed, we will document it and report it in a timely manner.

## Ethics and dissemination

This systematic review will be based on published data. As such, it is not subject to ethical approval. The results will be published in peer-reviewed journals and presented at scientific conferences. All data underlying the results will be made available upon reasonable request.

## Discussion

In the context of more available point of care echocardiography, TSPFO are expected to be increasingly reported among patients seen in the Emergency Department and intensive care unit for acute onset respiratory failure, shock or acute coronary syndromes ^7, 8^. The timely diagnosis of a TSPFO could radically influence acute treatment options and could provide critical information on potential patient outcomes. IS and MI can likely impact on the prognosis of patients with TSPFO, and are well-recognized complications of PFO in patients with pulmonary embolism and associated with increased risk of death ^9^. Paradoxical embolism to the coronary arteries has been reported less frequently and its incidence remains undetermined ^3^. Data on potential therapeutic interventions for patients with TSPFO are also scarce. A previous systematic review including 174 patients found a 35% lower 30-day mortality among surgically treated patients, although this was non-significant ^4^. Knowing the incidence of IS and MI in patients with TSPFO and their association with in-hospital death, as well as outcomes of different therapeutic interventions would be important for improving awareness about prognostic factors and treatment options in this population.

## Study status

Preliminary searches have been carried out in PubMed and Embase databases.

## Registration

This review protocol has been submitted to the International Prospective Register for Systematic Reviews and Meta-analysis (PROSPERO, registration number: CRD42020216118).

## Data availability

### Underlying data

No data is associated with this article.

### Reporting guidelines

Figshare: PRISMA-P checklist for ‘Determinants of in-hospital death in patients with a thrombus straddling a patent foramen ovale: protocol of a systematic review’, https://doi.org/10.6084/m9.figshare.13281242.v1 ^10^.

Data are available under the terms of the Creative Commons Attribution 4.0 International license (CC-BY 4.0).
