# Supplementary material for: Determinants of in-hospital death in patients with a thrombus straddling a patent foramen ovale: protocol of a systematic review
Source: F1000Res. 2021 May 5;9:1437. Originally published 2020 Dec 10. [Version 2] doi: 10.12688/f1000research.27622.2 (PMC8138489; doi:10.12688/f1000research.27622.2)
Supplement: Peer review history

# Peer review: Reviewer response for version 2

**DOI:** 10.5256/f1000research.56395.r84540

**Published:** 2021-05-20

**Authors:** Leonardo Varotto

**Affiliations:** 1 Department of Cardiology, San Bortolo Hospital, viale Rodolfi, Vicenza, 36100, Italy

I received your reply and understood your choices, even for not using the suggested technique. No need to review further your work, but I believe that its quality is currently not optimal because the a priori information is missing. Some steps you have taken are clear and I have no reason to doubt that it is good exploratory work. It is in fact impossible for me to judge at the moment the quantity and quality of the information you have access to.

Is the study design appropriate for the research question?

No

Is the rationale for, and objectives of, the study clearly described?

Yes

Are sufficient details of the methods provided to allow replication by others?

Yes

Are the datasets clearly presented in a useable and accessible format?

Yes

Reviewer Expertise:

Clinical trials, meta-analysis, interventional cardiology, interventional cardio-neurology

I confirm that I have read this submission and believe that I have an appropriate level of expertise to confirm that it is of an acceptable scientific standard.

# Peer review: Reviewer response for version 2

**DOI:** 10.5256/f1000research.56395.r84539

**Published:** 2021-05-19

**Authors:** Paolo Eusebi

**Affiliations:** 1 Regional Health Authority of Umbria, Perugia, Italy

Thanks for this revised version. The study protocol deserves indexing.

Is the study design appropriate for the research question?

Partly

Is the rationale for, and objectives of, the study clearly described?

Partly

Are sufficient details of the methods provided to allow replication by others?

Yes

Are the datasets clearly presented in a useable and accessible format?

Not applicable

Reviewer Expertise:

clinical trials; meta-analysis; diagnostics; data visualization

I confirm that I have read this submission and believe that I have an appropriate level of expertise to confirm that it is of an acceptable scientific standard.

# Peer review: Reviewer response for version 1

**DOI:** 10.5256/f1000research.30530.r80694

**Published:** 2021-03-29

**Authors:** Leonardo Varotto

**Affiliations:** 1 Department of Cardiology, San Bortolo Hospital, viale Rodolfi, Vicenza, 36100, Italy

- **Is the rationale for, and objectives of, the study clearly described?**

Yes, the importance of the study (the rationale that "promotes" it) is clear.

- **Is the study design appropriate for the research question?**

Partially or, more properly, no!

- **Are sufficient details of the methods provided to allow replication by others?**

Yes, with the limitations mentioned below.

- **Are the datasets clearly presented in a useable and accessible format?**

Yes, but this aspect cannot be currently fully judged.        

 Thank you for the opportunity to review this manuscript.

 From my point of view, the study design should better develop the following points that I would like to resume briefly as below:

- **1st problem:** The patients who will constitute the matrix and the data do not come from randomized studies, but from publications-observational studies and for this reason "pre-selected" in order to "promote a certain point of view" concerning, for example, a therapy, etc…
  Patient selection (not the selection made by the authors) constitutes a heavy source of bias.
  Is it not possible to conduct the study by aggregating multiple therapy centers (hospitals) with which to agree on a therapeutic and screening protocol? (see next point)…
- **2nd problem:** The search for co-morbidities, and in general the patient's health state and risk factors, is truly “refined” and timely. However, the absence of one of the information to be acquired in a specific patient does not mean that it was absent. Unfortunately, given that the patients do not come from the same study cohort with an a priori standardized observation protocol, it does not guarantee that the information collected is homogeneous.
  In the meta-analyzes conducted by aggregating randomized studies, this problem is addressed with graphic tools (funnel plot), or with in-depth analyzes on the heterogeneity of the studies.
  In summary, in this research plan (study design) not a word is spent on how to overcome these serious and typical problems of heterogeneity of patients connected to the purposes of the publication and to the different way in which they have been screened at the moment, for example, of hospitalization.
- **3rd problem:** In the data analysis techniques (logistic model) the search for interaction between the factors that could affect the probability of death in hospital of the patient does not appear and, moreover, using the "backward stepwise selection" strategy the first part of the univariate analysis becomes superfluous.
  It is not wrong to use a logistic regression model (although for such models it is always difficult to offer an overall picture of the quality of the obtained result) but I would also use an analysis technique such as the "random forest" which could probabilize patients with particular co-morbidities or risk factors.
  Of the logistic model, it is necessary to present also quality indicators: wrong classification, pseudo R2, Nagelkerke indicator. These are fundamental indicators to give credibility to the results obtained.
- **4th problem:** It is not clear how many patients could be analyzed. The author described a previous systematic review with 174 patients analysed, a number apparently very limited to contain measurement errors and to offer adequate power to statistical tests. What is the information gathering forecast?

I hope that the authors are encouraged by my remarks made with a constructive aim because it is very important to carry on such typology of studies. For this reason, it would be desirable that authors will accomplish their hard work with even more attention and dedication.

 To quote an aphorism: never give up. You would risk doing it an hour before the miracle!

Is the study design appropriate for the research question?

No

Is the rationale for, and objectives of, the study clearly described?

Yes

Are sufficient details of the methods provided to allow replication by others?

Yes

Are the datasets clearly presented in a useable and accessible format?

Yes

Reviewer Expertise:

Clinical trials, meta-analysis, interventional cardiology, interventional cardio-neurology

I confirm that I have read this submission and believe that I have an appropriate level of expertise to confirm that it is of an acceptable scientific standard, however I have significant reservations, as outlined above.

# Response

**Published:** 2021-04-22

**Authors:** Amado Jiménez-Ruiz

Dear Dr. Leonardo Varotto,

 First, we want to thank you for considering our manuscript and the time you and the reviewers devoted to reading it. We have received the feedback and suggestions, and we are addressing them. We have modified the manuscript according to the comments below.

 Impending paradoxical embolism is an uncommon condition rarely reported in the medical literature. Our pilot exercise for the review concluded that the published information is limited to case reports and small case series and therefore based our data extraction plan and further analysis on these results.

 During the process of analysis and selection of potential covariables for the project, we considered different methods. Some of those models were Ridge and LASSO. However, when we thought about our problem, we concluded that some variables should be in the model irrespective of their statistical significance (age and sex). In the case of LASSO and its autonomous nature for covariates for model selection, we could not ensure that the model would include both. Also, we performed multiple imputations through MICE; we cannot find a universal method to combine both techniques. For these two reasons, we selected a traditional stepwise approach.

 Instead of targeting significance for the covariates, we targeted overall accuracy in predictions or AIC.

 Thanks again for taking the time to review this manuscript.

 Sincerely,

 The Authors

# Peer review: Reviewer response for version 1

**DOI:** 10.5256/f1000research.30530.r80248

**Published:** 2021-03-08

**Authors:** Paolo Eusebi

**Affiliations:** 1 Regional Health Authority of Umbria, Perugia, Italy

- Why a meta-analysis of case reports and case series? Are other studies not available?
- Please correct covariable with covariate.
- Please consider adding penalized regressions like lasso (R package glmnet) in the methods. It could be a good option in the case of a small number of events per predictor.
- Please, consider cross-validation techniques.
- Instead of targeting significance for the covariates, it would be better to target overall accuracy in predictions or AIC.

Is the study design appropriate for the research question?

Partly

Is the rationale for, and objectives of, the study clearly described?

Partly

Are sufficient details of the methods provided to allow replication by others?

Yes

Are the datasets clearly presented in a useable and accessible format?

Not applicable

Reviewer Expertise:

clinical trials; meta-analysis; diagnostics; data visualization

I confirm that I have read this submission and believe that I have an appropriate level of expertise to confirm that it is of an acceptable scientific standard, however I have significant reservations, as outlined above.

# Response

**Published:** 2021-03-12

**Authors:** Amado Jiménez-Ruiz

Dear Dr. Paolo Eusebi,

 First, we want to thank you for considering our manuscript and the time you and the reviewers devoted to reading it. We have received the feedback and suggestions, and we are addressing them point by point. We have modified the manuscript according to the comments below.

***1. Why a meta-analysis of case reports and case series? Are other studies not available?***

 Impending paradoxical embolism is an uncommon condition rarely reported in the medical literature. Our pilot exercise for the review concluded that the published information is limited to case reports and small case series and therefore based our data extraction plan and further analysis on these results.

***2. Please correct covariable with covariate.***

 We have corrected covariable to covariate as stated.

***3. Please consider adding penalized regressions like lasso (R package glmnet) in the methods. It could be a good option in the case of a small number of events per predictor.***

 During the process of analysis and selection of potential covariables for the project, we considered different methods. Some of those models were Ridge and LASSO. However, when we thought about our problem, we conclude that some variables should be in the model irrespective of their statistical significance (age and sex). In the case of LASSO and its autonomous nature for covariates for model selection, we could not ensure that the model would include both. Also, we performed multiple imputations through MICE; we cannot find a universal method to combine both techniques. For these two reasons, we selected a traditional stepwise approach.

**4.** *Please, consider cross-validation techniques.*

 We appreciate the suggestion to use cross-validation. However, to manage uncertainty, we decided to perform sensitivity analysis using a multilevel model and the scenario of cases with complete data. We decided this because our target audience (cardiologists and neurologists) is more familiar with sensitivity analysis.

***5. Instead of targeting significance for the covariates, it would be better to target overall accuracy in predictions or AIC.***

 We appreciate the suggestion of using the AIC to select the best model. It is explicitly stated in the protocol now.

 Thanks again for taking the time to review this manuscript.

# Response

**Published:** 2021-04-22

**Authors:** Amado Jiménez-Ruiz

Dear Dr. Paolo Eusebi,

 First, we want to thank you for considering our manuscript and the time you and the reviewers devoted to reading it. We have received the feedback and suggestions, and we are addressing them point by point. We have modified the manuscript according to the comments below.

*Why a meta-analysis of case reports and case series? Are other studies not available?*

 Impending paradoxical embolism is an uncommon condition rarely reported in the medical literature. Our pilot exercise for the review concluded that the published information is limited to case reports and small case series and therefore based our data extraction plan and further analysis on these results.

 Please correct covariable with covariate.

 We have corrected covariable to covariate as stated.

*Please consider adding penalized regressions like lasso (R package glmnet) in the methods. It could be a good option in the case of a small number of events per predictor.*

 During the process of analysis and selection of potential covariables for the project, we considered different methods. Some of those models were Ridge and LASSO. However, when we thought about our problem, we conclude that some variables should be in the model irrespective of their statistical significance (age and sex). In the case of LASSO and its autonomous nature for covariates for model selection, we could not ensure that the model would include both. Also, we performed multiple imputations through MICE; we cannot find a universal method to combine both techniques. For these two reasons, we selected a traditional stepwise approach.

*Please, consider cross-validation techniques.*

 We appreciate the suggestion to use cross-validation. However, to manage uncertainty, we decided to perform sensitivity analysis using a multilevel model and the scenario of cases with complete data. We decided this because our target audience (cardiologists and neurologists) is more familiar with sensitivity analysis.

*Instead of targeting significance for the covariates, it would be better to target overall accuracy in predictions or AIC.*

 We appreciate the suggestion of using the AIC to select the best model. It is explicitly stated in the protocol now.

 Thanks again for taking the time to review this manuscript.

 Sincerely,

 The Authors
